# Sodium fluoride induces apoptosis through the downregulation of hypoxia-inducible factor-1α in primary cultured rat chondrocytes

**DOI:** 10.3892/ijmm.2013.1576

**Published:** 2013-12-05

**Authors:** HONGMEI MENG, TAO ZHANG, WEIDONG LIU, HUAN WANG, CHUNLEI WANG, ZHE ZHAO, NING LIU, WENBO WANG

**Affiliations:** 1 Department of Orthopedics, The First Affiliated Hospital of Harbin Medical University, Harbin, Heilongjiang 150000, P.R. China; 2 Department of Orthopedics, The First Hospital of Qiqihar, Qiqihar, Heilongjiang 161000, P.R. China

**Keywords:** sodium fluoride, chondrocyte, hypoxia-inducible factor 1α, sex determining region Y box gene 9, collagen II, apoptosis

## Abstract

It has been reported that sodium fluoride (NaF) suppresses the proliferation and induces apoptosis of chondrocytes. However, the cellular and molecular mechanisms of the effect have not been elucidated. Therefore, the aim of this study was to evaluate the mechanisms of the effects of NaF on primary cultured rat chondrocytes *in vitro*. Chondrocytes were treated with NaF at concentrations of 0, 1.5, 2.0, 2.5, 3.0, 3.5 and 4.0 mM. Cell viability decreased and the rate of apoptotic cells increased significantly with the gradient concentration of NaF in a time- and dose-dependent manner. Electron microscopy revealed cytoplasmic, organelle and nuclear alterations in the ultrastructure of chondrocytes exposed to various NaF concentrations. The cell cycle distribution was analyzed by flow cytometry, and the results indicated that NaF induced G2 cell cycle arrest. Western blotting was used to detect the apoptotic pathways. Downregulation of the Bcl-2 protein and upregulation of Bax, cleaved caspase-9, −12 and −3 proteins suggested that NaF was capable of inducing apoptosis through the mitochondrial and endoplasmic reticulum pathways. The results also showed that the levels of hypoxia-inducible factor 1α (HIF-1α), sex determining region Y box gene 9 (Sox9) and the collagen II (Col II) protein of the NaF groups were lower compared to those of the control groups. Thus, NaF may induce apoptosis through the downregulation of HIF-1α and disrupt the synthesis of extracellular matrix (ECM) through the downregulation of HIF-1α via the Sox9 pathway in primary cultured rat chondrocytes.

## Introduction

Fluoride is an essential trace element for human beings and animals. Exposure to fluoride takes the form of food, drinking water and burning coal (1–5). Appropriate amount of fluoride is beneficial to maintain bone strength and to protect against dental decay (6,7). However, long-term excessive intake of fluoride has deleterious effects on many organs and tissues including teeth, bone, liver, kidney and brain (8–18).

Results of previous studies have shown that fluoride has a higher and special affinity for calcium in bone tissue, which could result in osteofluorosis (19,20). It has been reported that osteofluorosis may increase the severity of osteoarthritis (OA) characterized by progressive degeneration of articular cartilage (21,22). Chondrocyte apoptosis may be involved in the onset and development of osteoarthritic cartilage degeneration (23). Previously, chondrocytes were found to be responsible for the synthesis of cartilage extracellular matrix (ECM) (24,25), while ECM is considered crucial to the survival of chondrocytes (26,27). Subsequently, there is a close correlation between chondrocyte apoptosis and the synthesis of ECM. Additionally, fluoride may induce OA by promoting chondrocytes apoptosis and disrupting the synthesis of ECM in cartilage; however, the molecular mechanism involved remains to be determined.

Hypoxia-inducible factor 1α (HIF-1α) is important in the maintenance of the survival of chondrocytes. HIF-1α may inhibit the apoptosis of chondrocytes and regulate the synthesis of ECM (28–30). This synthesis may be mediated by transactivation of sex determining region Y box gene 9 (Sox9), a key transcription factor for chondrocyte-specific genes such as collagen II (Col II) which encode the Col II protein (31,32). Thus, we hypothesized that sodium fluoride (NaF) would induce chondrocytes apoptosis through the downregulation of HIF-1α and cause matrix disruption through the downregulation of HIF-1α via the Sox9 pathway.

The purpose of this study was to demonstrate the molecular mechanism of NaF on apoptosis in primary cultured rat chondrocytes.

## Materials and methods

### Culture of primary rat chondrocytes

Chondrocytes were obtained from arthrodial cartilage of neonatal Wistar rats (Department of Laboratory Animal Science, The First Affiliated Hospital of Harbin Medical University, China). Cartilage was washed three times and minced in PBS with 100 IU/ml penicillin and 100 μg/ml streptomycin (Sigma, St. Louis, MO, USA). After digestion in 2.5 mg/ml trypsin for 40 min at 37°C, the cells were digested with 0.05 mg/ml collagenase type II (Gibco-BRL, Carlsbad, CA, USA) in DMEM/high glucose medium for 16 h at 37°C with 5% CO_2_ and passed through a 70-mm cell strainer to prepare a single-cell suspension. Approximately 1.5×10^5^ cells/ml were seeded in 25 cm^2^ culture flask containing DMEM/high glucose medium supplemented with 10% FBS, 100 IU/ml penicillin, and 100 μg/ml streptomycin and cultured at 37°C with 5% CO_2_ and 2% O_2_. After two days, cells adhering to the plate were passaged. The third or fourth generation was used in subsequent experiments.

### Identification of primary rat chondrocyte culture

Cells were grown for 1 day on glass coverslips coated with polylysine in DMEM/high glucose medium with 10% FBS in 6-well plates. The cells were fixed in cold 4% paraformaldehyde for 15 min at room temperature and made permeable with ice-cold 0.2% Triton X-100 in PBS for 8 min. Subsequent to blocking for 20 min with goat serum, primary antibody to Col II (Abcam, Cambridge, MA, USA) was applied at 1:50 overnight at 4°C. The cells were incubated for 1 h in secondary anti-rabbit antibody (Santa Cruz Biotechnology, Inc., Santa Cruz, CA, USA). Diaminobenzidine (DAB) was then used to detect positive staining (Zhongshan Biotechnology Co., Ltd., Beijing, China).

In order to observe the normal morphology of primary cultured rat chondrocytes, cells untreated with NaF were dyed with hematoxylin and eosin (H&E). Immunohistochemistry was used to identify the primary cultured rat chondrocytes by Col II protein.

### Analysis of cell viability

Cell viability was evaluated by MTT assay. A total of 3×10^3^ cells/well were plated in 96-well plates, and subsequent to overnight incubation, the cells were exposed to NaF for 24–72 h and washed with PBS. Fresh complete growth medium (1 ml) supplemented with 20 μl of MTT dye (0.5 mg/ml) was added to each well, and the mixture was incubated for 4 h at 37°C. Immediately after incubation, the cells were thoroughly washed with PBS. Dimethyl sulfoxide (DMSO) (0.5 ml/well) was added to stop the MTT reaction and to dissolve the formazan crystals. After agitation for 10 min at room temperature, the optical density in each well was detected at 490 nm with an ELISA reader (Bio-Rad, Hercules, CA, USA). Untreated chondrocytes were considered as 100% viable cells. The results were calculated using the formula: (OD treated well - OD blank)/(OD untreated well - OD blank) ×100%.

### Flow cytometric analysis of cell apoptosis

Cells (1×10^5^ cells/well) were seeded in 6-well plates and cultured overnight. Followin an increase in the concentration of NaF treatment, the cells were digested with 2.5 mg/ml trypsin and washed twice with PBS. The cells were suspended with 300 μl binding buffer, 2 μl Annexin V were added and the mixture was carefully agitated. Then, 5 μl propidium iodine (PI) (both from Baosai, Biotechnology, Beijing, China) was added to the mixture. Following incubation for 5–15 min at room temperature, the ratio of apoptosis was detected using a FACSCalibur flow cytometer (Becton-Dickinson, San Jose, CA, USA).

### Scanning electron microscopy

Cells were digested from the flask, centrifuged at 1500 rpm for 3 min to collect the cells, and fixed with 2.5% formaldehyde for 24 h at 4°C. Subsequently, the samples were fixed with 1% osmic acid for 1 h, dehydrated through a gradient ethanol series, embedded with epoxy resin and cut into sections. The sections were stained with uranyl acetate and lead citrate, observed and images were captured with a 1,200 electron microscope (Toshiba, Tokyo, Japan).

### Cell cycle analysis

Cells (2×10^5^ cells/well) were seeded in 6-well plates and starved in serum-free medium at 37°C. After 12-h starvation, the cells were treated with NaF solution and complete medium for 48 or 72 h. The cells were trypsinized and washed with PBS. Subsequently, the cells were resuspended with 500 μl RNase A (1 mg/ml) and PI (100 μg/ml) (Beyotime, Haimen, China) to stain the cell DNA. After 20-min incubation at room temperature in the dark, the DNA contents of the cells were analyzed using a FACSCalibur flow cytometer and the data were analyzed by ModFitLT V2.0 software (both from Becton-Dickinson).

### Western blot analysis

Cells were collected and total cell lysates were prepared using lysis buffer (Beyotime). Protein concentration was measured using the Bradford method with bovine serum albumin as the standard. Equal amounts (20–40 mg) of whole cell lysates were loaded into 8–12% SDS-polyacrylamide gels for electrophoresis at 80 V constant voltage and transferred to PVDF membranes (Millipore, Bedford, MA, USA) at 200 mA constant current for 3 h. The membrane was incubated in blocking buffer (5% skim milk in TBS-T) at room temperature for 2 h. The blocked membrane was then incubated with anti-HIF-1α (1:500), anti-Sox9 (1:500) (Abcam, Cambridge, UK), anti-Col II (1:250), anti-Bax (1:500), anti-Bcl-2 (1:500), anti-caspase-9 (1:500), anti-caspase-3 (1:500), (Santa Cruz Biotechnology, Inc.) and anti-caspase-12 (Biovision Inc., Mountain View, CA, USA) primary antibody at 4°C overnight and washed with TBS-T three times every 10 min, followed by incubation with a secondary antibody (1:1,000) (Santa Cruz Biotechnology, Inc.) at room temperature for 1 h. After washing in TBS-T, the immunoreactive bands were visualized using enhanced chemiluminescence kits (Pierce Biotechnology Inc., Rockford, IL, USA). Blots were stained with anti-β-actin or -GAPDH antibody (Santa Cruz Biotechnology, Inc.) as an internal control for the amounts of target proteins.

### Statistical analysis

Experiments were carried out three times. Data were presented as means ± SD. Differences among means were analyzed using the two-sided Student’s t-test or one-way ANOVA. P<0.05 was considered statistically significant.

## Results

### Morphology and identification of primary cultured rat chondrocytes

**Figure 1:**
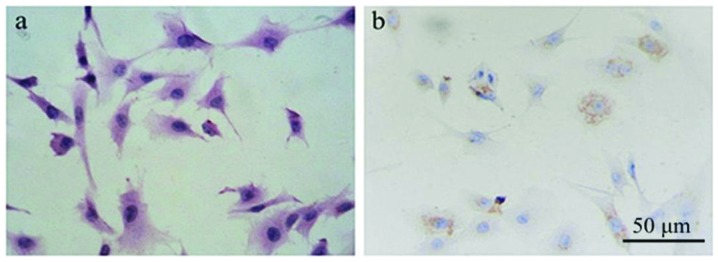
Chondrocytes were identified by collagen II (Col II) protein. (a) Hematoxylin and eosin (H&E) staining was used to detect the normal morphology of the primary cultured rat chondrocytes. Primary chondrocytes are polygonal, with a number of protrusions. (b) Immunohistochemistry was used to identify the primary cultured rat chondrocytes by Col II protein expressed in the cytoplasm.

In order to observe the normal morphology of primary cultured rat chondrocytes, cells untreated with NaF were dyed with H&E. As is shown in Fig. 1a, the primary chondrocytes are polygonal, with a number of protrusions, and connect to each other. Some secretory granules are also evident in the cytoplasm.

Col II is the chondrocyte-specific protein. Immunohistochemistry was used to identify rat chondrocytes by Col II protein (Fig. 1b). The results showed that the primary cultured chondrocytes expressed Col II protein in the cytoplasm, which is consistent with the characteristics of chondrocytes.

### Fluoride inhibits cell viability and causes changes in cell morphology in chondrocytes

**Figure 2:**
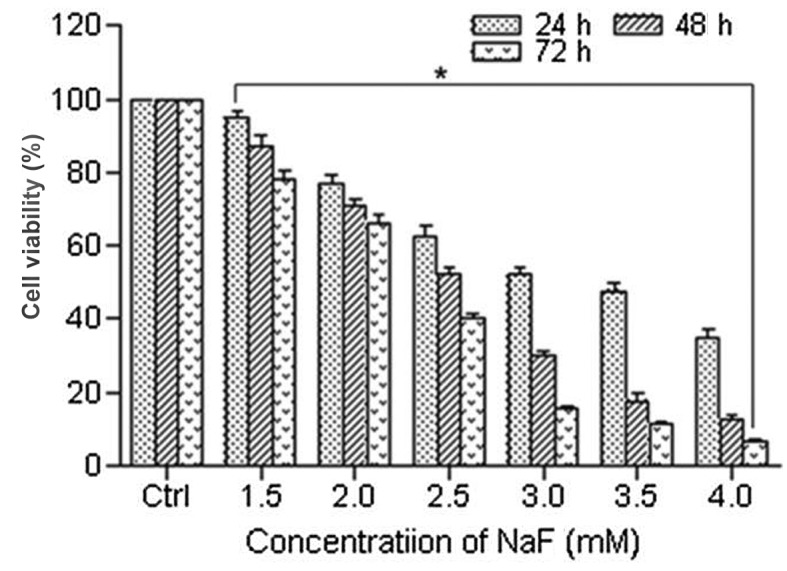
Sodium fluoride (NaF) inhibited cell viability of primary cultured rat chondrocytes. The effect of NaF on cell viability was measured by MTT assay. Cell viability of chondrocytes treated with gradient doses of NaF for 24, 48 and 72 h were decreased compared with that of the control groups, and were inhibited in a dose- and time-dependent manner. ^*^P<0.05 vs. control.

To evaluate the effect of NaF on chondrocytes, MTT assay was used to measure cell viability. Cells were treated with NaF at concentrations of 0, 1.5, 2.0, 2.5, 3.0, 3.5 and 4.0 mM for 24, 48 and 72 h. The viability of chondrocyte-exposed gradient doses of NaF (0, 1.5, 2.0, 2.5, 3.0, 3.5 and 4.0 mM) for 24, 48 and 72 h was (100±0, 94.90±1.92, 77.10±2.55, 62.36±3.16, 52.21±2.10, 47.81±2.17 and 34.72±2.86%); (100.00±0, 87.27±2.97, 70.95±1.94, 52.18±1.90, 30.11±1.41, 17.70±2.06 and 12.47±1.37%); (100.00±0, 77.92±2.52, 66.39±2.36, 40.22±1.41, 15.35±1.06, 11.51±0.82 and 6.70±0.52%) respectively. The cell viability of the NaF groups was significantly decreased compared with that of the control groups (Fig. 2). NaF significantly inhibited the viability of chondrocytes in a time- and dose-dependent manner.

**Figure 3:**
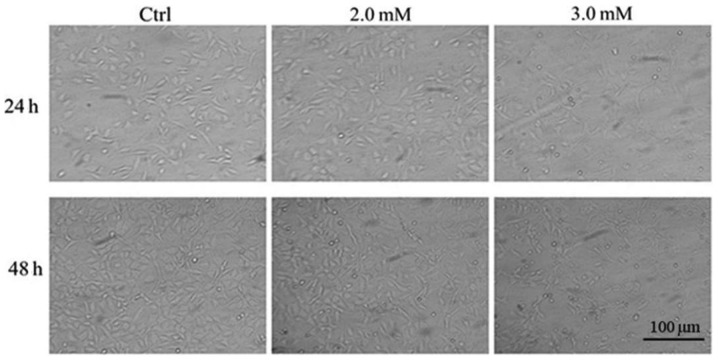
Sodium fluoride (NaF) caused a morphology change of chondrocytes. Phase-contrast images of chondrocytes before and after treatment with NaF at 0, 2.0 and 3.0 mM for 0, 24 and 48 h.

Morphological changes of chondrocytes following treatment with NaF at 2.0 and 3.0 mM for 24 and 48 h are shown in Fig. 3. Compared with NaF-untreated chondrocytes, chondrocytes treated with NaF (2.0 and 3.0 mM) became smaller, spindle-shaped and floated. These morphology changes demonstrated cell damage following NaF treatment.

### Analysis of cell apoptosis by Annexin V/PI staining

**Figure 4:**
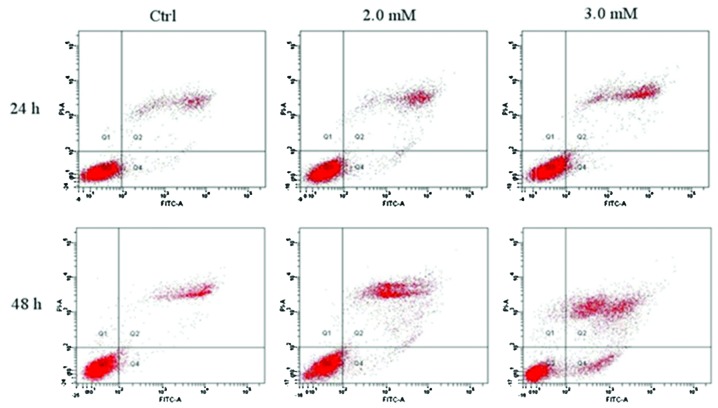
Flow cytometric analysis of Annexin V/PI was used to quantify sodium fluoride (NaF)-induced apoptosis in chondrocytes. Dot plots of chondrocytes with NaF treatment at 0, 2.0 and 3.0 mM for 24 or 48 h show an increase in the rate of apoptotic cells with increasing concentrations of NaF.

The rate of early apoptotic, late apoptotic or dead cells was analyzed with Annexin V/PI staining and flow cytometry. The apoptotic rate of chondrocytes treated with doses of NaF (2.0 and 3.0 mM) for 24 and 48 h was (17.28±2.27 and 24.53±1.36%) and (36.59±0.90 and 43.10±2.23%), respectively. The rates were significantly higher than those of the control groups (12.67±0.67%). As is shown in Fig. 4a and Table I, the rate of apoptotic cells increased significantly with increasing concentrations of NaF in a time- and dose-dependent manner.

### Ultrastructure observation

**Figure 5:**
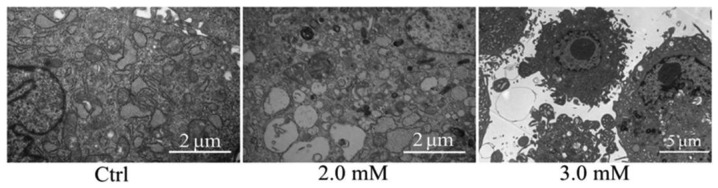
Ultrastructural changes of chondrocytes with sodium fluoride (NaF) treatment. Chondrocytes were treated with NaF at concentrations of 0, 2.0 and 3.0 mM for 48 h. Phase-contrast images of chondrocytes before and after treatment with NaF for 48 h showing that chromatin condensed into large clumps, while mitochondria and endoplasmic reticulum appear pale.

Chondrocytes were treated with NaF at 0, 2.0 and 3.0 mM for 48 h. The morphology of the control group was normal. Microvillis, irregular karyotype and evenly distributed chromatin were evident on the cell surface. Compared with the normal structure of the control group, the microvillis of the cell surface in the NaF groups were decreased, and the perinuclear cisterna was widened. Chromatin was condensed into large clumps, surrounding the nuclear membrane. Mitochondrias became swollen, degenerated and pale. The endoplasmic reticulum appeared pale, and the apoptotic cells appeared in NaF groups (Fig. 5).

### NaF induces cell cycle arrest in chondrocytes

**Figure 6:**
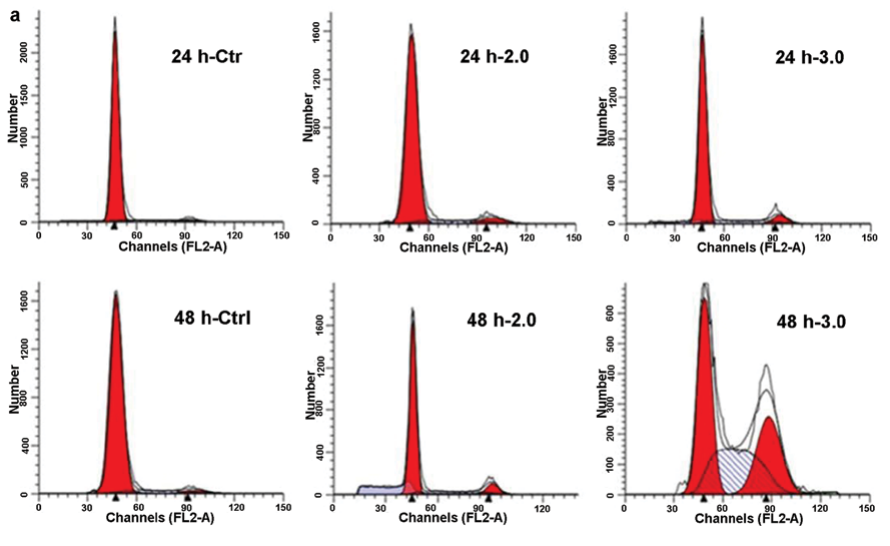

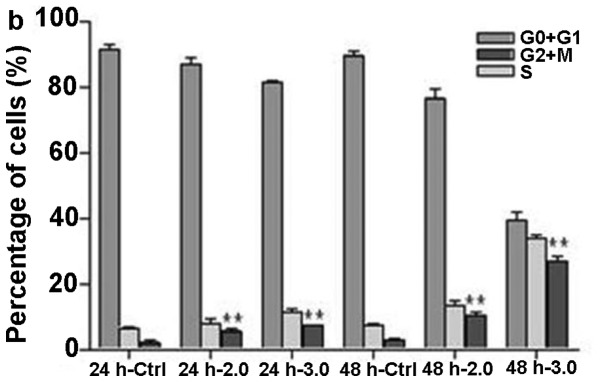
Cell cycle distribution of chondrocytes was analyzed by flow cytometry. (a) Arrest of cell cycle at G2 in chondrocytes in response to sodium fluoride (NaF) treatment. (b) The distribution of the cell cycle of chondrocytes was assessed by flow cytometry after staining with propidium iodide (PI). NaF significantly inhibited the proliferation of chondrocytes through cell cycle arrest at the G2 phase in a time- and dose-dependent manner. ^**^P<0.01 vs. control.

Cell cycle distribution of chondrocytes was analyzed by flow cytometry, aiming to determine whether the inhibitory effect was due to the cell cycle arrest. Chondrocytes were exposed to NaF at 2.0 and 3.0 mM for 24 and 48 h. Chondrocytes exposed to NaF showed G2 arrest by decreasing the fraction of G1 phase and increasing the fraction of G2 phase, as compared with that of the untreated cells (Fig. 6a and b). These results revealed that NaF arrested chondrocyte proliferation via cell cycle arrest at the G2 phase.

### Mechanisms of apoptosis after NaF treatment

**Figure 7:**
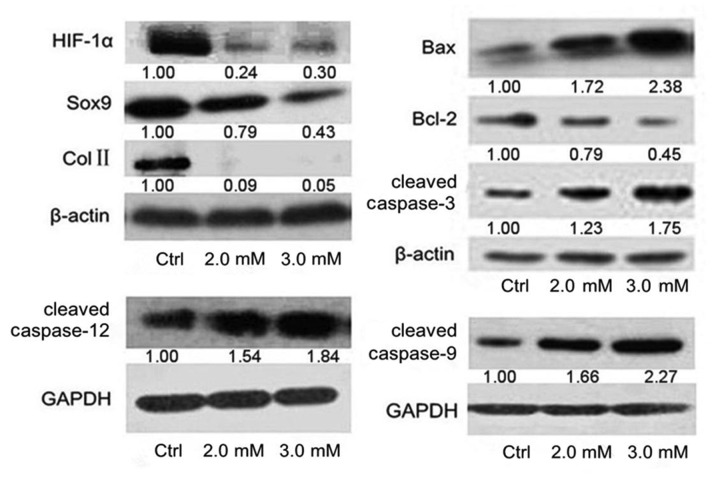
Effect of sodium fluoride (NaF) on hypoxia-inducible factor 1α (HIF-1α), sex determining region Y box gene 9 (Sox9), collagen II (Col II), Bcl-2, Bax, caspase-3, −9 and −12, β-actin and GAPDH protein expression in chondrocytes. Chondrocytes were exposed to NaF at concentrations of 0, 2.0 and 3.0 mM for 48 h. The expression of proteins was detected by western blotting. The relative amount of proteins expressed in chondrocytes was analysed.

To investigate the mechanisms of NaF causing apoptosis, the expression of HIF-1α, Sox9 and Col II, Bcl-2, Bax, caspase-9, −12 and −3 were assessed using western blotting (Fig. 7). Downregulation of Bcl-2 and upregulation of Bax, cleavaged caspase-9, −12 and −3 indicated that NaF induced apoptosis through the mitochondrial and endoplasmic reticulum pathways. The results also showed that the levels of HIF-1α, Sox9 and Col II protein of the NaF-treated groups were lower than those of control groups. Thus, NaF may induce apoptosis through the downregulation of HIF-1α and disrupt the synthesis of ECM through the downregulation of HIF-1α via Sox9 pathway in primary cultured rat chondrocytes.

## Discussion

OA, a degenerative joint disease, is the most common form of arthritis. The mainly affected peripheral joints are knees, hips and hands (33,34). The later clinic symptoms of OA include joint space stenosis, joint pains and the dysfunction of joint movement. Aging and mechanical overload have been considered as risk factors of OA, while excessive fluoride could increase the severity of OA (35,36). To the best of our knowledge, the effects of NaF on the degeneration of chondrocytes in OA have yet to be elucidated. In this study, NaF at concentrations of 1.5, 2.0, 2.5, 3.0, 3.5 and 4.0 mM was administered to primary cultured rat chondrocytes. The effects of NaF on the proliferation, apoptosis and synthesis of ECM in primary cultured rat chondrocytes were observed.

Ren *et al* have shown that primary cultured mouse osteoblasts treated with lower concentrations of NaF (10^−3^-1 mM) resulted in higher cell activities compared with control cells. However, the cell activities were significantly decreased at higher concentrations of NaF (5–10 mM) (37). In this study, the MTT assay indicated that NaF inhibited the cell activities of primary cultured rat chondrocytes with increasing concentration of NaF (1.5, 2.0, 2.5, 3.0, 3.5 and 4.0 mM) in a time- and dose-dependent manner. However, the effects of NaF on primary cultured mouse osteoblasts at lower concentrations of NaF (<1 mM) had no effects on primary cultured rat chondrocytes. This result may be due to cultivation conditions, incubation time and different cell lines. Results of the present study also showed the cell cycle distribution of chondrocytes in the subsequent experiments. According to the result of the MTT assay, chondrocytes were treated with NaF at concentrations of 2.0 and 3.0 mM in subsequent experiments. Our data showed a reduced number of chondrocytes in G1 and S phase and an increased number of chondrocytes in G2 phase in the NaF groups compared with the control groups. Additionally, the number of G2 phase cells gradually increased with the increasing in dose of NaF and treatment time. These results suggest that excessive NaF inhibited the proliferation of chondrocytes by inducing cell cycle arrest at G2 phase in a dose- and time-dependent manner. The result is concordant with a study by Wang *et al* (38). He and Chen (18) demonstrated that the number of rat oral mucosal cells and hepatocytes in G2/M phase was lower in NaF groups than that in control groups, however, there were no obvious changes in G0/G1 and S phase. The difference may be due to NaF having a different effect on the cell cycle in different types of cells.

Findings of previous studies have shown that excessive NaF can trigger apoptosis in different types of cells including osteoblasts, epithelial lung cells, sperm cells, leukocytes and ameloblasts (38–42). In the present study, the rate of apoptosis was assessed by Annexin V/PI staining and flow cytometric analysis. The results showed the rate of apoptosis in NaF groups was significantly increased compared with that of the control groups, and that it gradually increased with the increasing NaF concentrations and treatment time. This suggests that NaF induced chondrocyte apoptosis in a dose- and time-dependent manner. Furthermore, the ultrastructure of chondrocytes was detected by transmission electron microscopy. The results revealed that the control groups possessed integrated cell membrane, abundant cytoplasm, evenly distributed chromatin, a large number of abnormal mitochondrias and well-developed endoplasmic reticulum. However, the NaF groups had various degenerative biological characteristics including swelling of mitochondria, dilation of endoplasmic reticulum, reduced electron dense material, chromatin condensation and gathered chromatin at the nuclear periphery.

The ultrastructural changes suggest that NaF has adverse effects on chondrocytes through the mitochondrial and endoplasmic reticulum pathways. In order to demonstrate whether NaF was actually capable of inducing apoptosis through the mitochondrial and endoplasmic reticulum pathways in primary rat chondrocytes, apoptotic markers, including Bax, Bcl-2, caspase-9, −12 and −3 were detected by western blotting. In this study, the level of Bcl-2 protein in the NaF groups was significantly lower than that of the control groups, while the levels of Bax, cleavaged caspase-9, −12 and −3 proteins were significantly higher than those of the control groups. These data confirmed that NaF was capable of inducing the apoptosis of chondrocytes through the mitochondrial and endoplasmic reticulum pathways, although the mechanism of NaF on apoptosis in chondrocytes remains to be clarified.

HIF-1α is known to play a key role in the development and progression of articular cartilage degeneration in OA. It can maintain the cartilage homeostasis by regulating the downstream genes involved in the proliferation, apoptosis and synthesis of ECM components in chondrocytes (28–30). This synthesis may, at least partly, be mediated by the transactivation of Sox9, a key transcription factor for cartilage-specific marker genes such as Col II and aggrecan (31,32). Yudoh *et al* (43) reported that HIF-1α-deficient chondrocytes showed significantly increased levels of apoptosis compared with the control groups. This observation suggests that a decreased expression of HIF-1α may have the ability to promote chondrocyte apoptosis. In the present study, the level of HIF-1α protein was significantly decreased with increasing concentrations of NaF. In other words, NaF may induce cell apoptosis by inhibiting the expression of HIF-1α protein in primary cultured rat chondrocytes. Moreover, in order to investigate the possible molecular mechanisms of NaF-induced matrix disruption in chondrocytes, the expression of Sox9 and Col II proteins were assessed *in vitro*. The level of Col II protein was significantly decreased along with the decreasing Sox9 protein. Thus, NaF may inhibit the synthesis of Col II protein through the downregulation of HIF-1α via the Sox9 pathway in primary cultured rat chondrocytes. It appears that there is a stong correlation between the NaF-induced apoptosis and synthesis of ECM in chondrocytes. Chondrocyte apoptosis may disrupt matrix synthesis. Conversely, matrix disruption also affects the survival of chondrocytes.

Taken together, results of the present study suggest that NaF inhibits the synthesis of Col II protein through downregulation of HIF-1α via Sox9 pathway and induces cell apoptosis through the downregulation of HIF-1α expression in primary cultured chondrocytes. The apoptotic pathway included endoplasmic reticulum intrinsic and mitochondrial intrinsic pathways. However, future studies should be conducted to clarify the exact mechanisms involved.

## Figures and Tables

**Table I:** Percentage of quadrant distribution (QD) in Annexin V/PI staining apoptosis assay.

	24 h			48 h		
		
Variables	Control	2.0 NaF	3.0 NaF	Control	2.0 NaF	3.0 NaF
Necrosis	0.20±0.17	0.25±0.26	0.13±0.06	0.50±0.17	0.55±0.38	1.87±0.25^b^
Early apoptosis	2.07±0.64	3.10±0.17	4.13±0.61^a^	1.63±0.21	4.62±1.11^b^	7.70±1.06^b^
Late apoptosis	10.60±1.01	14.18±2.12	20.40±1.85^a^	11.20±1.42	31.97±0.24^b^	35.40±2.87^b^
Normal	87.13±0.93	82.51±2.54	75.37±1.36^a^	86.63±1.38	62.82±1.05^b^	55.37±1.50^b^

a P<0.05,

b P<0.01 vs. control.

## References

[1] Malde MK, Scheidegger R, Julshamn K, Bader HP (2011). Substance flow analysis: a case study of fluoride exposure through food and beverages in young children living in Ethiopia. Environ Health Perspect.

[2] Pandey J, Pandey U (2011). Fluoride contamination and fluorosis in rural community in the vicinity of a phosphate fertilizer factory in India. Bull Environ Contam Toxicol.

[3] Hussain I, Arif M, Hussain J (2012). Fluoride contamination in drinking water in rural habitations of Central Rajasthan, India. Environ Monit Assess.

[4] Xiao YH, Sun F, Li CB, Shi JQ, Gu J, Xie C, Guan ZZ, Yu YN (2011). Effect of endemic fluoride poisoning caused by coal burning on the oxidative stress in rat testis (In Chinese). Zhongguo Yi Xue Ke Xue Yuan Xue Bao.

[5] Li HL, Yu YN, Chen Y, Huang L (2012). Effect of fluoride on oxidative stress and Mn-SOD expression in rats with endemic fluorosis of coal burning (In Chinese). Zhonghua Bing Li Xue Za Zhi.

[6] Gutiérrez-Salinas J, Morales-González JA, Madrigal-Santillán E, Esquivel-Soto J, Esquivel-Chirino C, González-Rubio MG, Suástegui-Domínguez S, Valadez-Vega C (2010). Exposure to sodium fluoride produces signs of apoptosis in rat leukocytes. Int J Mol Sci.

[7] Ricomini Filho AP, Tenuta LM, Fernandes FS, Calvo AF, Kusano SC, Cury JA (2012). Fluoride concentration in the top-selling Brazilian toothpastes purchased at different regions. Braz Dent J.

[8] Vieira AP, Hanocock R, Eggertsson H, Everett ET, Grynpas MD (2005). Tooth quality in dental fluorosis genetic and environmental factors. Calcif Tissue Int.

[9] Denbesten P, Li W (2011). Chronic fluoride toxicity: dental fluorosis. Monogr Oral Sci.

[10] Whyte MP, Essmyer K, Gannon FH, Reinus WR (2005). Skeletal fluorosis and instant tea. Am J Med.

[11] Bezerra de Menezes LM, Volpato MC, Rosalen PL, Cury JA (2003). Bone as a biomarker of acute fluoride toxicity. Forensic Sci Int.

[12] Wang AG, Xia T, Chu QL, Zhang M, Liu F, Chen XM, Yang KD (2004). Effects of fluoride on lipid peroxidation, DNA damage and apoptosis in human embryo hepatocytes. Biomed Environ Sci.

[13] Shanthakumari D, Srinivasalu S, Subramanian S (2004). Effect of fluoride intoxication on lipidperoxidation and antioxidant status in experimental rats. Toxicology.

[14] Santoyo-Sanchez MP, del Carmen Silva-Lucero M, Arreola-Mendoza L, Barbier OC (2013). Effects of acute sodium fluoride exposure on kidney function, water homeostasis, and renal handling of calcium and inorganic phosphate. Biol Trace Elem Res.

[15] Basha PM, Madhusudhan N (2010). Pre and post natal exposure of fluoride induced oxidative macromolecular alterations in developing central nervous system of rat and amelioration by antioxidants. Neurochem Res.

[16] Seraj B, Shahrabi M, Shadfar M, Ahmadi R, Fallahzadeh M, Eslamlu HF, Kharazifard MJ (2012). Effect of high water fluoride concentration on the intellectual development of children in makoo/iran. J Dent (Tehran).

[17] Cicek E, Aydin G, Akdogan M, Okutan H (2005). Effects of chronic ingestion of sodium fluoride on myocardium in a second generation of rats. Hum Exp Toxicol.

[18] He LF, Chen JG (2006). DNA damage, apoptosis and cell cycle changes induced by fluoride in rat oral mucosal cells and hepatocytes. World J Gastroenterol.

[19] Levy SM, Eichenberger-Gilmore J, Warren JJ, Letuchy E, Broffitt B, Marshall TA, Burns T, Willing M, Janz K, Torner JC (2009). Associations of fluoride intake with children’s bone measures at age 11. Community Dent Oral Epidemiol.

[20] Chachra D, Vieira AP, Grynpas MD (2008). Fluoride and mineralized tissues. Crit Rev Biomed Eng.

[21] Savas S, Cetin M, Akdoğan M, Heybeli N (2001). Endemic fluorosis in Turkish patients: relationship with knee osteoarthritis. Rheumatol Int.

[22] Nishida T, Kubota S, Aoyama E, Takigawa M (2013). Impaired glycolytic metabolism causes chondrocyte hypertrophy-like changes via promotion of phospho-Smad1/5/8 translocation into nucleus. Osteoarthritis Cartilage.

[23] Goggs R, Carter SD, Schulze-Tanzil G, Shakibaei M, Mobasheri A (2003). Apoptosis and the loss of chondrocyte survival signals contribute to articular cartilage degradation in osteoarthritis. Vet J.

[24] Blanco FJ, Guitian R, Vázquez-Martul E, de Toro FJ, Galdo F (1998). Osteoarthritis chondrocytes die by apoptosis. A possible pathway for osteoarthritis pathology. Arthritis Rheum.

[25] Hashimoto S, Ochs RL, Komiya S, Lotz M (1998). Linkage of chondrocyte apoptosis and cartilage degradation in human osteoarthritis. Arthritis Rheum.

[26] Peters HC, Otto TJ, Enders JT, Jin W, Moed BR, Zhang Z (2011). The protective role of the pericellular matrix in chondrocyte apoptosis. Tissue Eng Part A.

[27] Thomas CM, Fuller CJ, Whittles CE, Sharif M (2007). Chondrocyte death by apoptosis is associated with cartilage matrix degradation. Osteoarthritis Cartilage.

[28] Provot S, Schipani E (2007). Fetal growth plate: a developmental model of cellular adaptation to hypoxia. Ann NY Acad Sci.

[29] Schipani E, Ryan HE, Didrickson S, Kobayashi T, Knight M, Johnson RS (2001). Hypoxia in cartilage: HIF-1alpha is essential for chondrocyte growth arrest and survival. Genes Dev.

[30] Pfander D, Cramer T, Schipani E, Johnson RS (2003). HIF-1alpha controls extracellular matrix synthesis by epiphyseal chondrocytes. J Cell Sci.

[31] Kypriotou M, Fossard-Demoor M, Chadjichristos C, Ghayor C, de Crombrugghe B, Pujol JP, Galéra P (2003). SOX9 exerts a bifunctional effect on type II collagen gene (COL2A1) expression in chondrocytes depending on the differentiation state. DNA Cell Biol.

[32] Apichart V, Wong R, Rabie B, Lei S (2012). The effect of quercetin on expression of SOX9 and subsequent release of type II collagen in spheno-occipital synchondroses of organ-cultured mice. Angle Orthod.

[33] Andrianakos AA, Kontelis LK, Karamitsos DG, Aslanidis SI, Georgountzos AI, Kaziolas GO, Pantelidou KV, Vafiadou EV, Dantis PC, The ESORDIG study (2006). Prevalence of symptomatic knee, hand, and hip osteoarthritis in Greece. J Rheumatol.

[34] Bennell K, Hinman RS, Wrigley TV, Creaby MW, Hodges P (2011). Exercise and osteoarthritis: cause and effects. Compr Physiol.

[35] Zhang Y, Niu J, Kelly-Hayes M, Chaisson CE, Aliabadi P, Felson DT (2002). Prevalence of symptomatic hand osteoarthritis and its impact on functional status among the elderly: The Framingham Study. Am J Epidemiol.

[36] Horisberger M, Fortuna R, Valderrabano V, Herzog W (2013). Long-term repetitive mechanical loading of the knee joint by in vivo muscle stimulation accelerates cartilage degeneration and increases chondrocyte death in a rabbit model. Clin Biomech (Bristol, Avon).

[37] Ren G, Ferreri M, Wang Z, Su Y, Han B, Su J (2011). Sodium fluoride affects proliferation and apoptosis through insulin-like growth factor I receptor in primary cultured mouse osteoblasts. Biol Trace Elem Res.

[38] Wang Z, Yang X, Yang S, Ren G, Ferreri M, Su Y, Chen L, Han B (2011). Sodium fluoride suppress proliferation and induce apoptosis through decreased insulin-like growth factor-I expression and oxidative stress in primary cultured mouse osteoblasts. Arch Toxicol.

[39] Thrane EV, Refsnes M, Thoresen GH, Låg M, Schwarze PE (2001). Fluoride-induced apoptosis in epithelial lung cells involves activation of MAP kinases p38 and possibly JNK. Toxicol Sci.

[40] Sun Z, Niu R, Wang B, Jiao Z, Wang J, Zhang J, Wang S, Wang J (2011). Fluoride-induced apoptosis and gene expression profiling in mice sperm in vivo. Arch Toxicol.

[41] Yan Q, Zhang Y, Li W, Denbesten PK (2007). Micromolar fluoride alters ameloblast lineage cells in vitro. J Dent Res.

[42] Qu WJ, Zhong DB, Wu PF, Wang JF, Han B (2008). Sodium fluoride modulates caprine osteoblast proliferation and differentiation. J Bone Miner Metab.

[43] Yudoh K, Nakamura H, Masuko-Hongo K, Kato T, Nishioka K (2005). Catabolic stress induces expression of hypoxia-inducible factor (HIF)-1 alpha in articular chondrocytes: involvement of HIF-1 alpha in the pathogenesis of osteoarthritis. Arthritis Res Ther.

